# Systematic Analysis of Pericarp Starch Accumulation and Degradation during Wheat Caryopsis Development

**DOI:** 10.1371/journal.pone.0138228

**Published:** 2015-09-22

**Authors:** Xurun Yu, Bo Li, Leilei Wang, Xinyu Chen, Wenjun Wang, Zhong Wang, Fei Xiong

**Affiliations:** 1 Jiangsu Key laboratory of Crop Genetics and Physiology/Co-Innovation Center for Modern Production Technology of Grain Crops, Yangzhou University, Yangzhou 225009, China; 2 Jiangsu Yanjiang Institute of Agricultural Sciences, Nantong 226541, China; Saint Mary's University, CANADA

## Abstract

Although wheat (*Triticum aestivum* L.) pericarp starch granule (PSG) has been well-studied, our knowledge of its features and mechanism of accumulation and degradation during pericarp growth is poor. In the present study, developing wheat caryopses were collected and starch granules were extracted from their pericarp to investigate the morphological and structural characteristics of PSGs using microscopy, X-ray diffraction and Fourier transform infrared spectroscopy techniques. Relative gene expression levels of ADP-glucose pyrophosphorylase (*APGase*), granule-bound starch synthase II (*GBSS II*), and α-amylase (*AMY*) were quantified by quantitative real-time polymerase chain reaction. PSGs presented as single or multiple starch granules and were synthesized both in the amyloplast and chloroplast in the pericarp. PSG degradation occurred in the mesocarp, beginning at 6 days after anthesis. Amylose contents in PSGs were lower and relative degrees of crystallinity were higher at later stages of development than at earlier stages. Short-range ordered structures in the external regions of PSGs showed no differences in the developing pericarp. When hydrolyzed by α-amylase, PSGs at various developmental stages showed high degrees of enzymolysis. Expression levels of *AGPase*, *GBSS II*, and *AMY* were closely related to starch synthesis and degradation. These results help elucidate the mechanisms of accumulation and degradation as well as the functions of PSG during wheat caryopsis development.

## Introduction

Wheat is one of the most important cereal crops cultivated worldwide. Wheat caryopsis contains an abundance of nutrients, including starch, proteins, minerals, vitamins, and phytochemicals [1]. Starch is the major stored carbohydrate in wheat caryopsis and accounts for approximately 65% to 75% of caryopsis dry weight [2]. Wheat caryopsis consists of the germ (embryo and scutellum), endosperm, and pericarp [3]. The pericarp, which lies in the outermost layers of the caryopsis, is composed of three layers, namely, the epicarp, mesocarp, and endocarp [4]. During wheat caryopsis development, starch granules are mainly synthesized in the endosperm and pericarp within a double-membrane-bound organelle called the plastid [5]. Two types of plastids have been identified: the amyloplast, in which starch granules are synthesized and accumulate for long periods of time, and the chloroplast, in which photosynthesis takes place and transitory starch is synthesized [6]. Transitory starch is considered both an overflow for newly assimilated carbon and storage for carbohydrates during periods of darkness when photosynthesis is impossible. Transitory starch usually appears in non-storage tissues and organs, such as the pericarp and leaves, in higher plants [7, 8].

**Fig 1:**
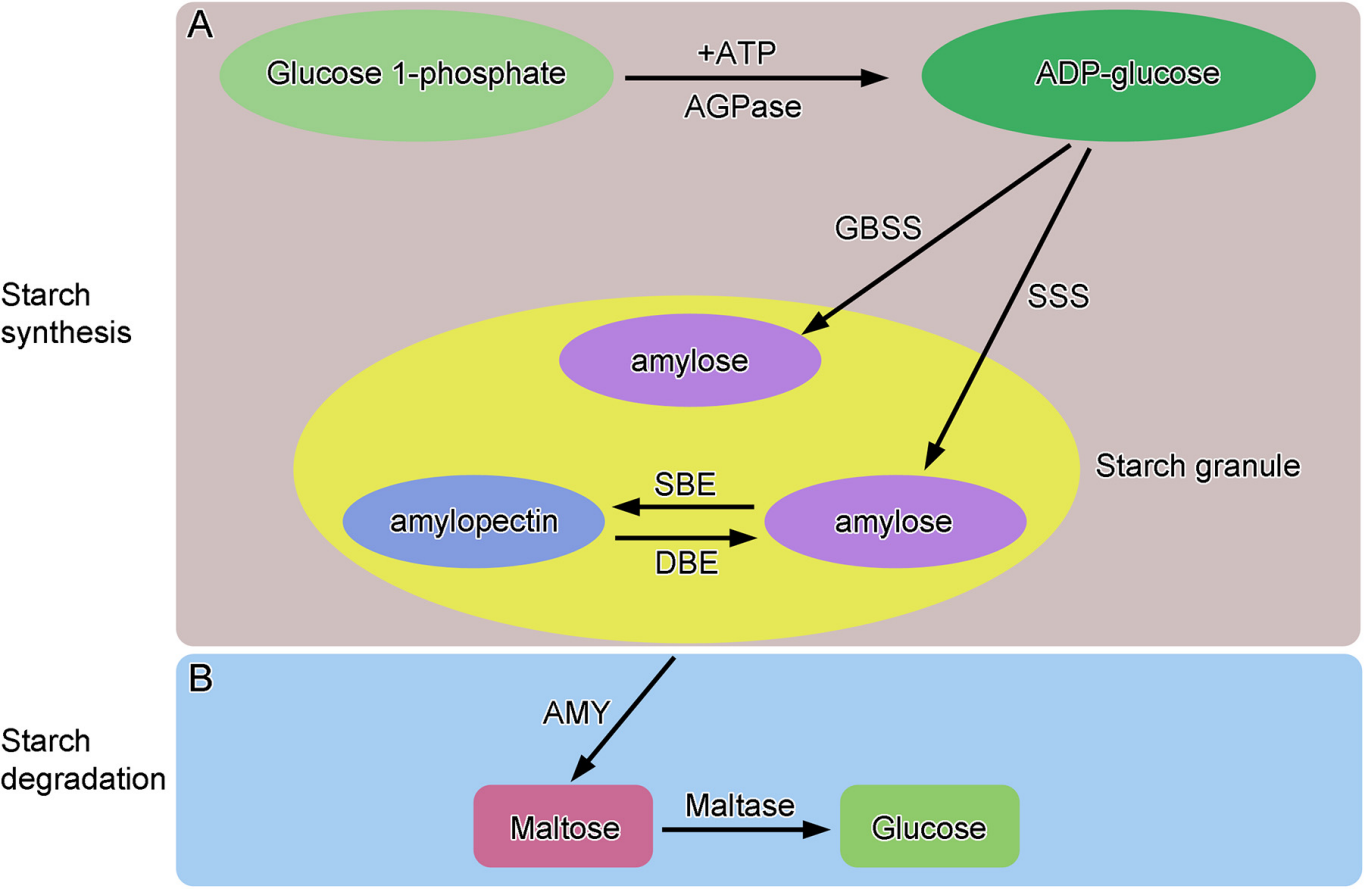
Starch synthesis (A) and degradation (B) pattern. *AGPase* ADP-glucose pyrophosphorylase; AMY, α-amylase; DBE, starch debranching enzyme; GBSS, granule-bound starch synthase; SBE, starch branching enzyme; SSS, soluble starch synthase.

Starch is composed of two major glucose polymers, namely, amylose and amylopectin, which differ in their degree of polymerization of glucan chains [9]. The key enzymes involved in starch biosynthesis (Fig 1) are ADP-glucose pyrophosphorylase (AGPase), granule-bound starch synthase (GBSS), soluble starch synthase (SSS), starch branching enzyme (SBE) and starch debranching enzyme (DBE). AGPase is the key enzyme bringing about the first step of starch synthesis. GBSS is responsible for amylose synthesis in plant organs. Two isoforms of GBSS exist: GBSS I, which exists in storage organs, such as parenchymal cells of potato tubers and caryopsis endosperm cells of Gramineae crops, and GBSS II, which can only be detected in non-storage organs or tissues, such as the pericarp, leaf, and stem [5, 10]. In the process of wheat seed germination, α-amylase (AMY) catalyzes starch hydrolysis into maltose. Thereafter, maltose is hydrolyzed into glucose by maltase (Fig 1).

In cereal crop endosperm cells, starch granules are synthesized in the amyloplast and increase in number and volume until caryopsis maturity [11, 12]. The morphological and physicochemical properties of starch granules change during each phase of endosperm development in cereal crops, such as rice and wheat [13, 14]. However, unlike endosperm starch granules, pericarp starch granules (PSGs) exhibit dynamic changes during caryopsis development. Several reports concerning the development of PSGs of Gramineae crops have been published. PSGs are spherical in shape at the early stages of maize kernel development [6–12 days after anthesis (DAA)] but become irregular and polygonal at later stages (14–30 DAA). By contrast, amylose contents, amylopectin structures, and the thermal properties of PSGs remain similar across different developmental stages [15]. Starch granules in *Sorghum bicolor* (L.) pericarp consist of multiple starch granules; they present high accumulations in the mesocarp at early stages of caryopsis development but eventually disappear at maturity [7]. In rice caryopsis pericarp, starch granules accumulate in plastids in exterior cells and accumulate in chloroplasts in inner cells, including cross cells, but gradually degrade as caryopsis development proceeds [16]. In wheat pericarp, starch granules are synthesized in amyloplasts or chloroplasts from anthesis to 11 DAA but gradually disappear after 11 DAA [17]. Similar results have been reported by Yu et al. (2015) [18], who pointed out that PSGs exhibit a typical accumulation peak at 5 DAA and then gradually decompose until 22 DAA. Xiong et al. (2013) [19] studied the structural and physiological features of wheat pericarp development and found that PSGs accumulate in mesocarp cells at 3 DAA, reach maximum accumulation at 9 DAA, and completely disappear after 15 DAA.

Although the accumulation characteristics of PSGs are well-studied, other characteristics, such as starch granule development in different pericarp regions as well as the structural characteristics and physicochemical properties of PSGs in developing wheat caryopsis, remain poorly understood. Moreover, our knowledge of the amylose content, relative expression of the related synthetic and degradation enzyme genes (*AGPase*, *GBSS II*, and *AMY*), and degradation mechanisms of PSGs remains limited. In our previous study, the development and physicochemical properties of PSGs and endosperm starch granules were compared [18]. Based on those findings, dynamic changes in PSGs were systematically analyzed at the morphological, structural, physiological, physicochemical, and molecular levels in the present study. Our results provide novel insights into the functions of PSGs and their mechanisms of accumulation and degradation during wheat caryopsis development. Furthermore, our findings hold broad implications for the fields of plant development, plant physiology, and molecular biology.

## Materials and Methods

### Plant material

The high-yielding hard winter wheat (*Triticum aestivum* L. cv. Yannong 19) seeds used in the present study were purchased from the National Wheat Improvement Center (Yangzhou, China) and grown in the experimental field of Yangzhou University (32°39'N, 119°42'E), Key Laboratory of Crop Genetics and Physiology of Jiangsu Province from September 2013 to May 2014. The field soil was sandy loam (Typic fluvaquents, Entisols (US taxonomy)) that contained 24.5 g kg^-1^ organic materials as well as 106, 33.8, and 66.4 mg kg^-1^ of available N, P, and K, respectively. The climate condition represented a transition from humid subtropical monsoon climate to temperate monsoon climate. The average annual temperature was 15.2°C and the annual rainfall was 961–1048 mm. The plants received normal fertilizer and water supply during growing stages. To determine the anthesis date accurately, individual florets were marked at the base of the spikelet in the middle of spikes and whole plants were tagged.

### Starch granule extraction

Fresh wheat caryopses were collected 2, 6, 10, and 20 DAA and kept at –20°C until starch extraction. The pericarp was separated from the caryopsis by hand and immediately immersed in absolute ethyl alcohol to inactivate its enzymes. Starch granules were extracted from the pericarp following the method of Yu et al. (2015) [18] with slight modifications. The separated pericarp was ground with 0.2% NaOH solution in a mortar, and the ground sample was filtered through 12 gauze layers. The filtrate was then centrifuged at 2,500 ×*g* for 5 min at 25°C, and the yellow green gel containing the protein and tissue fragments was scraped from the starch granules. This step was repeated four to five times until no more gel could be obtained. Finally, the starch samples were washed twice with distilled water and absolute ethyl alcohol and dried at 40°C for 48 h.

### Determination of total starch, soluble sugar, and amylose contents

The separated pericarp was dried in an oven at 110°C for 3 h and completely ground in a mortar. The powder obtained was used to determine total starch and soluble sugar contents via total starch (QY1-1; Comin Biotechnology Co., Ltd., China) and soluble sugar (SY3-2; Comin Biotechnology Co., Ltd.) kits. Amylose contents in the extracted starch were determined using an amylose determination kit (K-AMYL; Megazyme Ltd., Ireland).

### Structural observation

Caryopses were collected 2, 6, 10, and 20 DAA and cut breadthwise into 2 mm-thick slices from the center. Samples were immediately immersed in 0.1 M glutaraldehyde-phosphate buffer fixative (pH 7.2) at 4°C for 3 h and then fixed with 0.5% OsO_4_ for 3 h. Fixed samples were subsequently rinsed, dehydrated, infiltrated, and embedded according to the method described by Zheng and Wang (2011) [20].

For light microscopy, samples were cut into 1 μm slices using an ultramicrotome (Ultracut R, Leica, Germany), stained with 0.5% methyl violet for 10 min, and photographed under a light microscope (DMLS, Leica, Germany).

For transmission electron microscopy, samples were cut into 60 nm slices using an ultramicrotome (Ultracut R, Leica, Germany), respectively stained with uranyl acetate and lead citrate for 30 and 15 min, and photographed under a transmission electron microscope (Tecnai 12, Philips, Holland).

For fluorescence microscopy, caryopses were collected and cut breadthwise into 1 mm-thick slices from the center. Samples were placed on a microslide and observed and photographed under a fluorescence microscope (DMLB, Leica, Germany) equipped with a digital camera (Power Shot S50, Canon, Japan).

For scanning electron microscopy, 1 mg of starch was mixed with 500 μL of absolute ethyl alcohol. The mixture (20 μL) was placed in a stage groove and dried at 40°C for 1 h. Samples were then gold-sputtered under vacuum and photographed using a scanning electron microscope (SEM; S4800, Hitachi, Japan). The accelerating voltage and current of the instrument were 15 kV and 10 μA, respectively. The size distribution of starch granules in the micrographs was analyzed using Image-Pro Plus software (Version 6.0, Media Cybernetics, USA). A total of 200 starch granules were combined into one sample, and the means of three replicate samples (600 total) were used for calculations.

### X-ray diffraction (XRD) analysis

XRD was used to investigate the crystalline structure of starch granules including the crystalline type and relative degree of crystallinity. Prior to determination, starch samples were stored in a closed container containing 100 mL of saturated NaCl solution maintained under a constant-humidity atmosphere (relative humidity = 75%) for 7 d. Wet starch samples were placed on a vitric sample platform and scanned at diffraction angles (coupled 2θ) from 4° to 40° with a step size of 0.2 s. The ratio of the crystallinity area to the total diffraction area was calculated as the relative degree of crystallinity (%) from the XRD patterns obtained using Image-Pro Plus software (Version 6.0, Media Cybernetics, USA) [21].

### Attenuated total reflectance Fourier transform infrared (ATR-FTIR) spectroscopy analysis

An FTIR spectrometer equipped with an attenuated total reflectance (ATR) system was used to analyze ordered structures in the external region of the starch granules [22]. Starch (30 mg) was mixed with 25 μL of distilled water, and the resulting slurry was placed on the sample platform of an FTIR spectrometer (Varian 7000, Varian, USA). FTIR spectra were recorded from 1200 cm^−1^ to 800 cm^−1^ and then deconvoluted with a resolution enhancement factor of 1.9 and a half-width of 19 cm^−1^ [23]. Infrared (IR) absorbance bands at 1045, 1022, and 995 cm^−1^ were measured by recording the height of the bands from baseline to peak point.

### Starch enzymolysis

Starch was hydrolyzed using porcine pancreatic α-amylase (ECC 3.2.1.1; Sigma–Aldrich A3176). Starch samples (10 mg) were suspended in 2 mL of enzyme solution (0.1 M sodium phosphate buffer, pH 6.9, 25 mM NaCl, 5 mM CaCl_2_, 0.02% NaN_3_, and 50 U PPA) [24]. Hydrolysis was implemented in a constant-temperature shaking water bath at 37°C for 0.5, 1, 2, 4, 8, 12, and 24 h. After hydrolysis, samples were centrifuged at 3,000 ×*g* for 10 min at 4°C. To determine the degree of hydrolysis, soluble sugar contents in the supernatant were measured using a soluble sugar kit (SY3-2; Comin Biotechnology Co., Ltd.).

### RNA isolation, cloning, and sequencing

The pericarp was separated from the caryopsis by hand at 2, 6, 10, and 20 DAA. The separated pericarp was immediately frozen in liquid nitrogen for 5 min and then stored at –70°C until RNA isolation. Total RNA was extracted from the pericarp using an *RNAprep* pure plant kit (Tiangen-bio, Beijing, China). Each sample (5 mg) was reverse transcribed into cDNA using the PrimeScript RT reagent kit (TaKaRa, Dalian, China) following the manufacturer’s instructions. PCR amplifications were performed using primer pairs (Table 1) to obtain the open reading frames of *AGPase*, *GBSS II*, and *AMY* genes. The PCR conditions were as follows: 95°C for 5 min; 30 cycles of 94°C for 30 s, 54°C for 30 s, and 72°C for 60 s; and 72°C for 10 min. The product was ligated into a *pMD*-18 vector (*TaKaRa*) and used to transform the *E*. *coli* strain DH5α. After PCR identification of the extracted plasmid, DNA sequencing was performed using GenScript (Nanjing, China) (**S1 Table**).

**Table 1:** Sequences of specific primers used for PCR amplification and qPCR.

Gene	Forward primer 5’-3’	Reverse primer 5’-3’
*AGPase*	CGGCAATGGATGTGCCTTTGGCATC	GCTTCATATGACTGTTCCACTAGG
*AGPase*-qPCR	GTACTTGTGGCTATTCGAGGAGCATAA	AAGGCAGCAACAGTAATATCAGCATCT
*GBSSⅡ*	ATGGGTTCCATTCCTAATTATTG	TCAGGGAGTGGCCACATTCTGT
*GBSSⅡ*-qPCR	CCTCACATCCAATTCCAGCAATCTTG	GCATTCCAGTTGAACATACGATAGGTG
*AMY*	GGCAAGCACTCTGCTACTCTCTG	TCAGAGGCCGCTCTTCTCCCAGAC
*AMY*-qPCR	GCCTGGCTCAACTGGCTCAA	ACGAACGACGGCTTGCTGTT

### Relative quantitative real-time polymerase chain reaction (qPCR) analysis

qPCR analysis was carried out on a qPCR system (ABI 7500, Applied Biosystems, USA) with SYBR Premix Ex Taq (TaKaRa). Wheat Ta2291 (*ADP-ribosylation factor* gene), which is considered one of the most stably expressed genes [25], was used as the reference gene and amplified together with target genes. The primers used for Ta2291 were 5’-AGTACAAGAACATCAGCTTCACTGTCT-3’ and 5’-CGTAACTCATCCTCATTCAGCATCCT-3’. qPCR primers were designed based on the high identity of the three gene nucleotide acid sequences (Table 1). The PCR conditions were as follows: 95°C for 30 s; 40 cycles of 95°C for 10 s and 54°C for 30 s, and 65°C for 15 s. Relative gene expression was calculated according to Pfaffl (2001) [26].

### Statistical analysis

Data were statistically analyzed using SPSS Statistics (Version 19.0, International Business Machines Corp., USA). Means were compared using Fisher’s protected least significant difference test at the 0.05 probability significance level.

## Results

### Accumulation and degradation of starch granules in different regions of the pericarp

**Fig 2:**
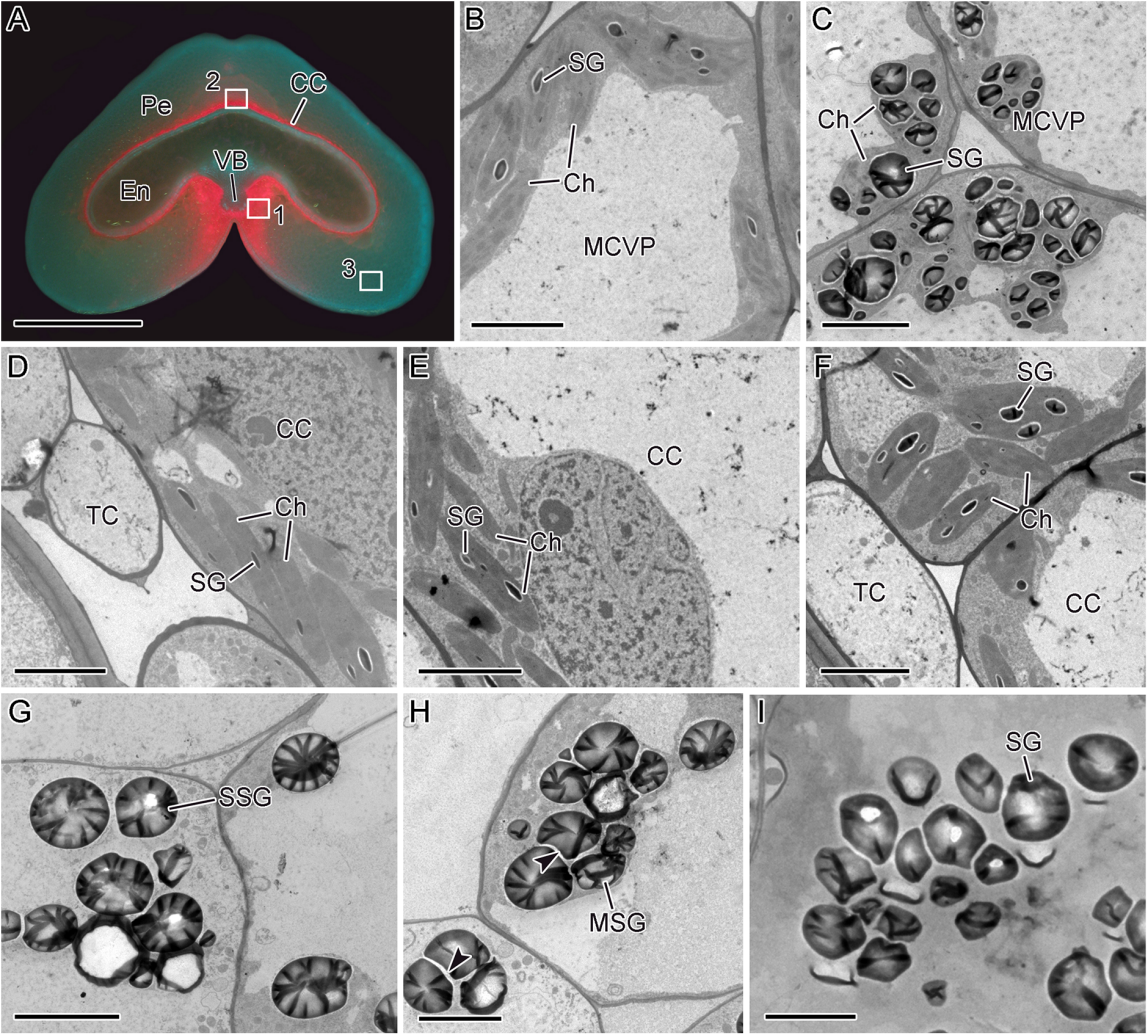
Fluorescence (A) and transmission electron microscope (B–I) images. Transverse section of wheat caryopsis at 6 DAA (**A**); higher magnification of the white box 1 in **A** at 6 DAA (**B**); higher magnification of white box 1 in **A** at 20 DAA (**C**); higher magnification of white box 2 in **A** at 6 DAA (**D, E**); higher magnification of white box 2 in **A** at 20 DAA (**F**); higher magnification of white box 3 in **A** at 6 DAA (**G, H**); higher magnification of white box 3 in **A** at 20 DAA (**I**). CC, cross cell; Ch, chloroplast; MSG, multiple starch granule; En, endosperm; MCVB, mesocarp cell near vascular bundle; Pe, pericarp; SG, starch granule; SSG, single starch granule; TC, tube cell; VB, vascular bundle. Scale bars: 1 mm (**A**) and 5 μm (**B–I**).

Fig 2A shows images of transverse sections of wheat caryopsis acquired using a fluorescence microscope. Red fluorescence was observed in mesocarp cells near the vascular bundle (MCVB) and cross cells, which indicates the presence of chloroplasts in these cells. At 6 DAA, small elongated starch granules were synthesized in chloroplasts in the MCVB and cross cell (Fig 2B, 2D and 2E). However, in mesocarp cells, starch granules were larger in size, spherical and irregular in shape, and synthesized in amyloplasts near the cell wall(Fig 2G and 2H). Starch granules presented as single or multiple granules, and spaces between granules could be observed clearly(Fig 2H). At 20 DAA, the number and volume of starch granules increased both in the MCVB and cross cells (Fig 2C and 2F). In the MCVB, chloroplasts increased in volume, and 4–7 starch granules were found (Fig 2C). These starch granules were spherical and irregular in shape. In cross cells, 1–4 starch granules were synthesized, and these granules appeared elongated in shape (Fig 2F). At the same time, starch granules in the mesocarp separated from each other and were freed within the cell (Fig 2I).

**Fig 3:**
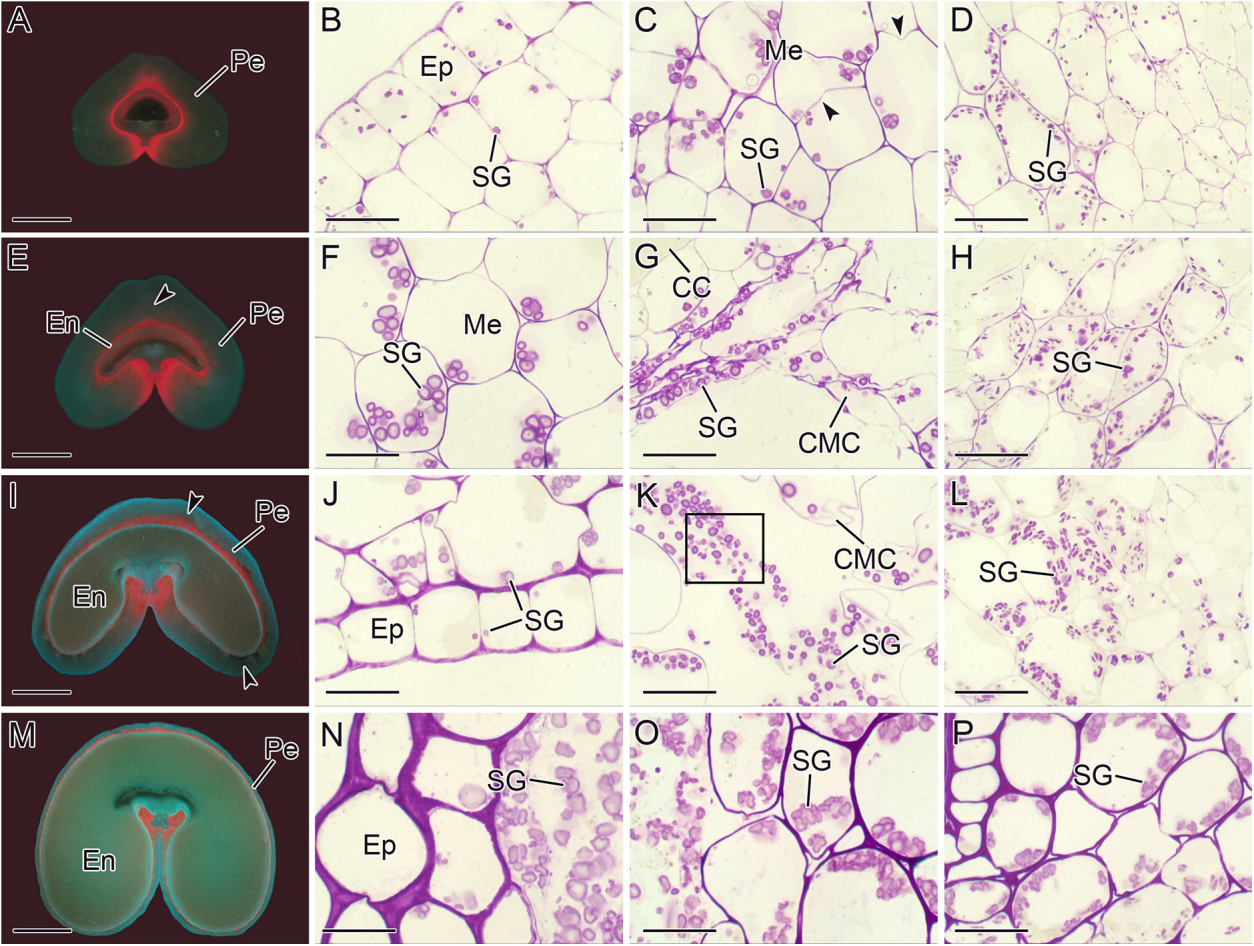
Fluorescence microscopy images of transverse sections of wheat caryopsis at 2 DAA (A), 6 DAA (E), 10 DAA (I), and 20 DAA (M). Light microscopy images of wheat pericarp (**B–D, F–H, J–L,** and **N–P**): epicarp (**B**), mesocarp (**C**), and mesocarp near the vascular bundle (**D**) at 2 DAA; mesocarp (**F, G**) and mesocarp near the vascular bundle (**H**) at 6 DAA; epicarp (**J**), mesocarp (**K**), and mesocarp near the vascular bundle (**L**) at 10 DAA; epicarp (**N**) and mesocarp near the vascular bundle (**O** and **P**) at 20 DAA. Black arrowheads in **C** and **E, I** indicate new cell walls and apoptotic cavities. The black box in **K** indicates degraded starch granules in the mesocarp. CC, cross cell; CMC, cracked mesocarp cell; En, endosperm; Ep, epicarp; Me, mesocarp; Pe, pericarp; SG, starch granule. Scale bars: 1 mm (**A, E, I,** and **M**) and 20 μm (**B–D, F–H, J–L,** and **N–P**).

At 2 DAA, a large number of starch granules accumulated in different regions of the pericarp, including the epicarp, mesocarp, and MCVB (Fig 3A, 3B, 3C and 3D). New cell walls were clearly observed in mesocarp cells (arrowheads in Fig 3C), which indicates active cell division.

At 6 DAA, apoptosis cavities (black arrowhead in Fig 3E) appeared in the mesocarp, especially in mesocarp cells adjacent to cross cells (Fig 3G), and starch granules were released from cracked mesocarp cells. In the mesocarp located far from cross cells, starch granules were larger than those observed at 2 DAA (Fig 3F).

At 10 DAA, a large number of apoptosis cavities were observed in most parts of the mesocarp (black arrowheads in Fig 3I). These were sites where starch granules had completely degraded. Incompletely degraded granules were also seen (black box in Fig 3K). In the epicarp, the number of starch granules at 10 DAA was less than that at 2 DAA (Fig 3J).

At 20 DAA, a thin layer of pericarp was observed in the outermost layer of endosperm (Fig 3M). At this time, two cell layers, namely the epicarp and mesocarp, were visible. No starch granules could be observed in the epicarp, and some residual starch granules that had been freed within the apoptosis cavities were seen (Fig 3N). Unlike starch granules in other parts of the pericarp, starch granules in the MCVP presented a remarkably different feature: they had not degraded but instead increased in size (Fig 3D, 3H, 3L, 3O and 3P). It could also be observed that the size of endosperm tissues increased much more than that of caryopsis (Fig 3A, 3E, 3I and 3M).

### Morphology and amylose content of PSGs during wheat pericarp development

**Fig 4:**
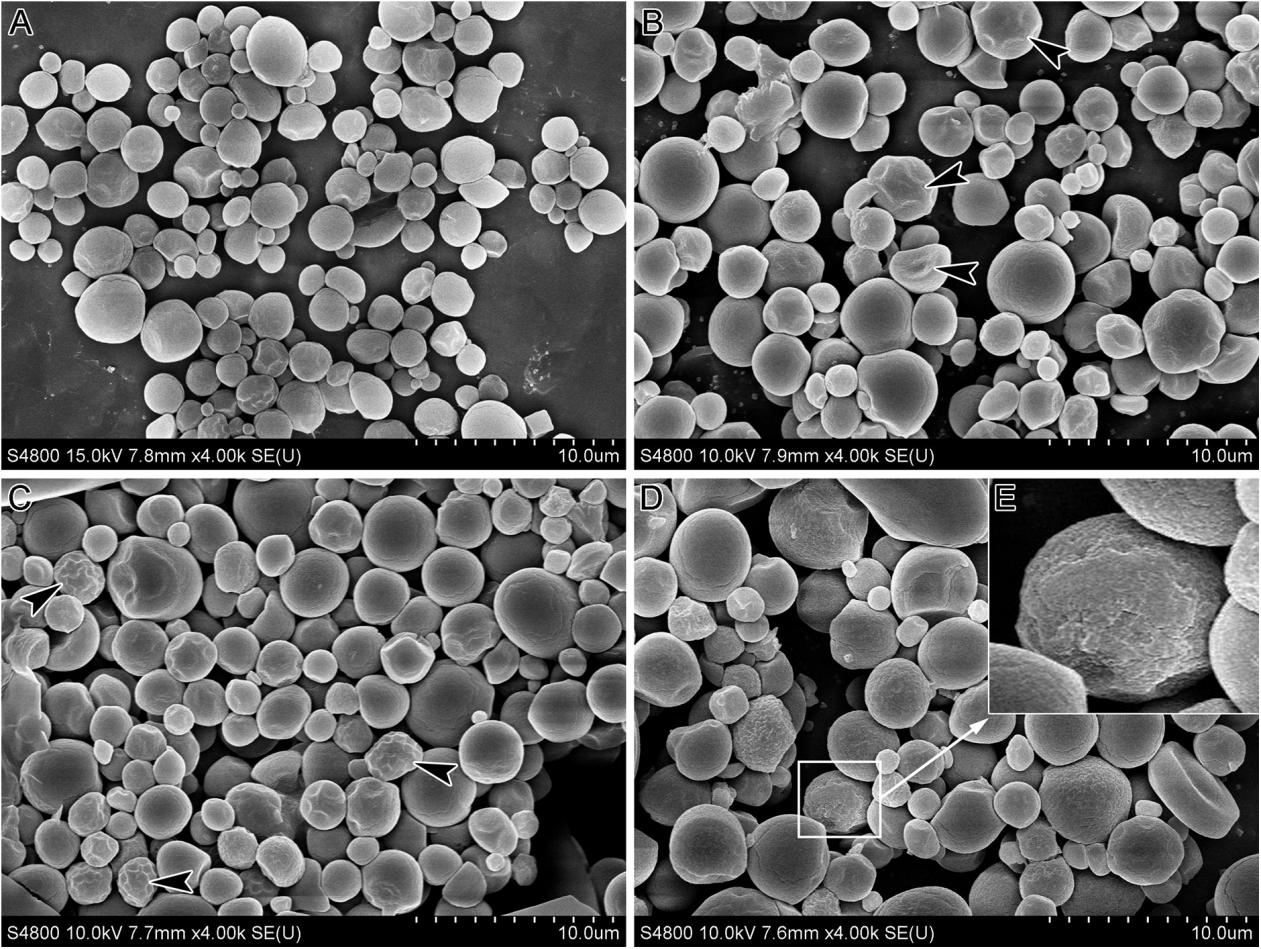
SEM images of PSGs at 2 DAA (A), 6 DAA (B), 10 DAA (C) and 20 DAA (D); higher magnification of the white box in d (E). Black arrowheads in **B** and **D** indicate polyhedral, irregular, and collapsed starch granules.

From 2 DAA to 20 DAA, starch granules gradually increased in size distribution from 0.64–4.80 μm to 0.76–8.02 μm, with the corresponding average sizes increasing from 1.98 μm to 2.74 μm (Table 2). SEM images of starch granules are shown in Fig 4.

**Table 2:** Size distribution, amylose content, relative degree of crystallinity, and IR ratio of PSGs at 2, 6, 10, and 20 DAA.

DAA	Minimum size (μm)	Maximum size (μm)	Average size (μm)	Amylose content (%)	Relative degree of crystallinity (%)	1045/1022 cm^-1^	1022/995 cm^-1^
2	0.64±0.06a	4.80±0.15a	1.98±0.12a	28.09±0.22a	29.84±0.39a	0.66±0.01a	1.48±0.01a
6	0.65±0.10a	4.85±0.07a	2.26±0.08b	27.86±0.08a	29.43±0.14a	0.67±0.01a	1.40±0.02b
10	0.79±0.01b	5.95±0.24b	2.47±0.06c	25.55±0.34b	31.39±0.24b	0.67±0.00a	1.41±0.01bc
20	0.76±0.02b	8.02±0.59c	2.74±0.05d	25.69±0.19b	31.11±0.09b	0.67±0.01a	1.36±0.01d

Values in the same column with different lowercase letters are significantly different (P ≤ 0.05).

At 2 DAA, starch granules were spherical and irregular in shape (Fig 4A). At 6 DAA, some polyhedral and collapsed starch granules could be found in granule groups (black arrowheads in Fig 4B). At 10 DAA, more polyhedral starch granules were observed (black arrowheads in Fig 4C). Starch granules were larger at 20 DAA than at previous phases (2, 6, and 10 DAA). Interestingly, a large number of starch granules with rough surfaces and apparent corrosion were also observed (Fig 4D and 4E).

The amylose contents of PSGs at 2, 6, 10, and 20 DAA are listed in Table 2. Amylose contents were significantly higher at 2 and 6 DAA than at 10 and 20 DAA.

### Changes in total starch and soluble sugar contents in developing wheat pericarp

**Fig 5:**
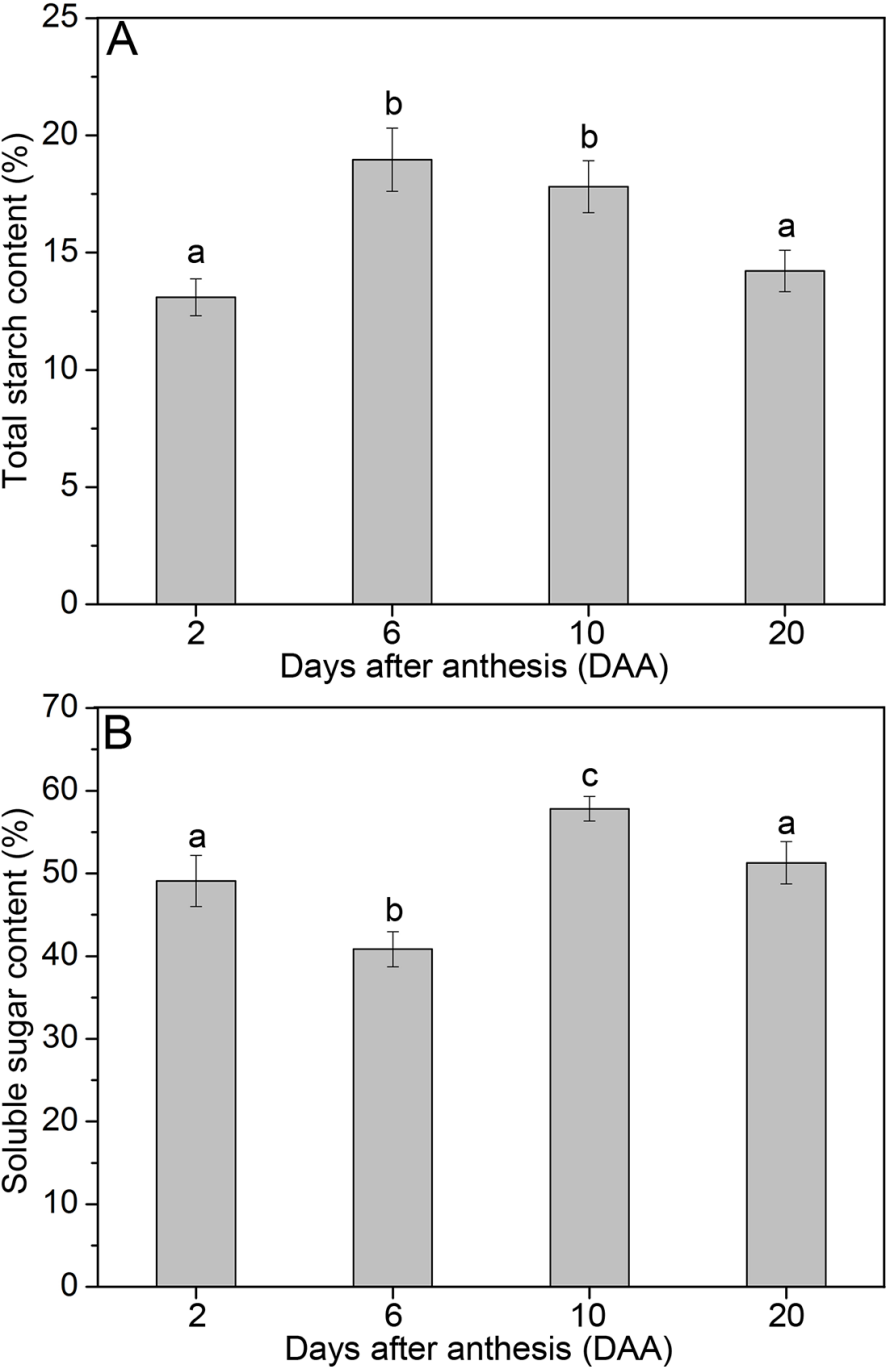
Total starch (A) and soluble sugar (B) contents of wheat pericarp at 2, 6, 10, and 20 DAA. Data represent the means of three replicates, and standard deviations are shown as vertical bars. Significant differences (P ≤ 0.05) between different DAA values are indicated by lowercase letters.

Fig 5 shows the total starch and soluble sugar contents of the pericarp at 2, 6, 10, and 20 DAA. Total starch contents initially increased from 2 DAA to 6 DAA and then decreased from 6 DAA to 20 DAA (Fig 5A). By contrast, soluble sugar contents first decreased, then increased, and finally decreased (Fig 5B). These changes in soluble sugar content are remarkably different from the changes observed in total starch content.

### XRD and ATR-FTIR analyses of PSG

**Fig 6:**
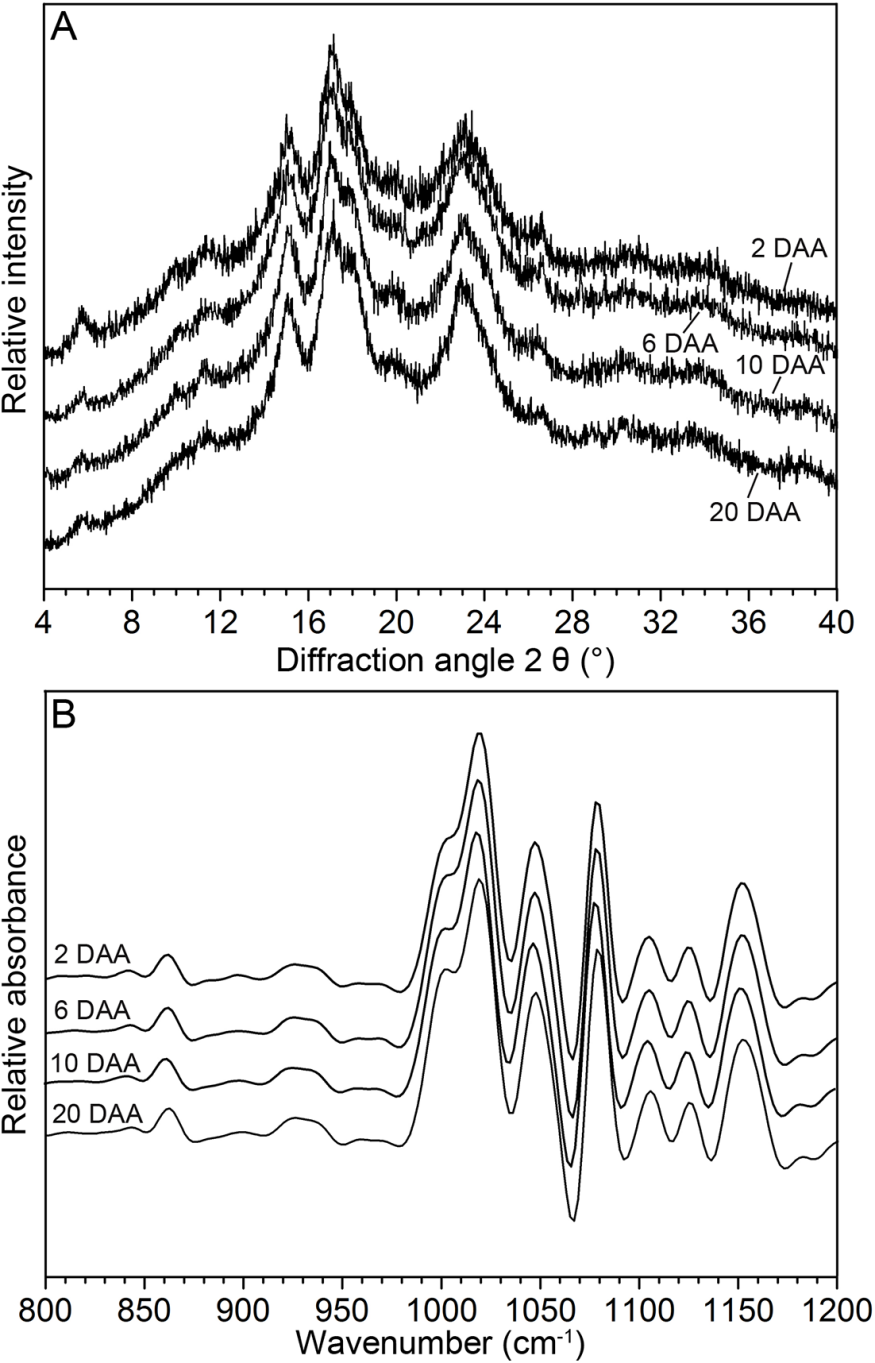
XRD (A) and ATR-FTIR (B) spectra of PSGs at 2, 6, 10, and 20 DAA.

The XRD spectra of PSGs recorded from 2θ of 4° to 40° are shown in Fig 6A. The PSGs presented strong reflections at 2θ of 15°, 17°, 18°, and 23° at 2, 6, 10, and 20 DAA, respectively. The spectra obtained were characterized by A-type crystal structures. Relative degrees of crystallinity were calculated using the appropriate software, and the results are listed in Table 2. Relative degrees of crystallinity were significantly higher at 10 and 20 DAA than at 2 and 6 DAA.

Amylose and short chains of amylopectin formed a double helix structure (long-range ordered stucture). Meanwhile, these double-helical molecules held the crystalline stucture (short-range ordered stucture) within starch granules via intermolecular interaction forces. PSGs at different developmental stages showed similar ATR-FTIR spectra (Fig 6B). No significant differences in the IR ratio of 1045 cm^−1^/1022 cm^−1^ were observed (Table 1). The IR ratio of 1022 cm^−1^/995 cm^−1^ was highest at 2 DAA and lowest at 20 DAA, followed by that at 6 and 10 DAA. XRD analysis further indicated that long-range ordered structures observed throughout the entire granules were more prominent at later developmental stages (10 and 20 DAA) than earlier stages (2 and 6 DAA). By contrast, short-range ordered structures in the external region of the granules did not exhibit changes with pericarp development.

### Enzymolysis degree of PSGs

**Fig 7:**
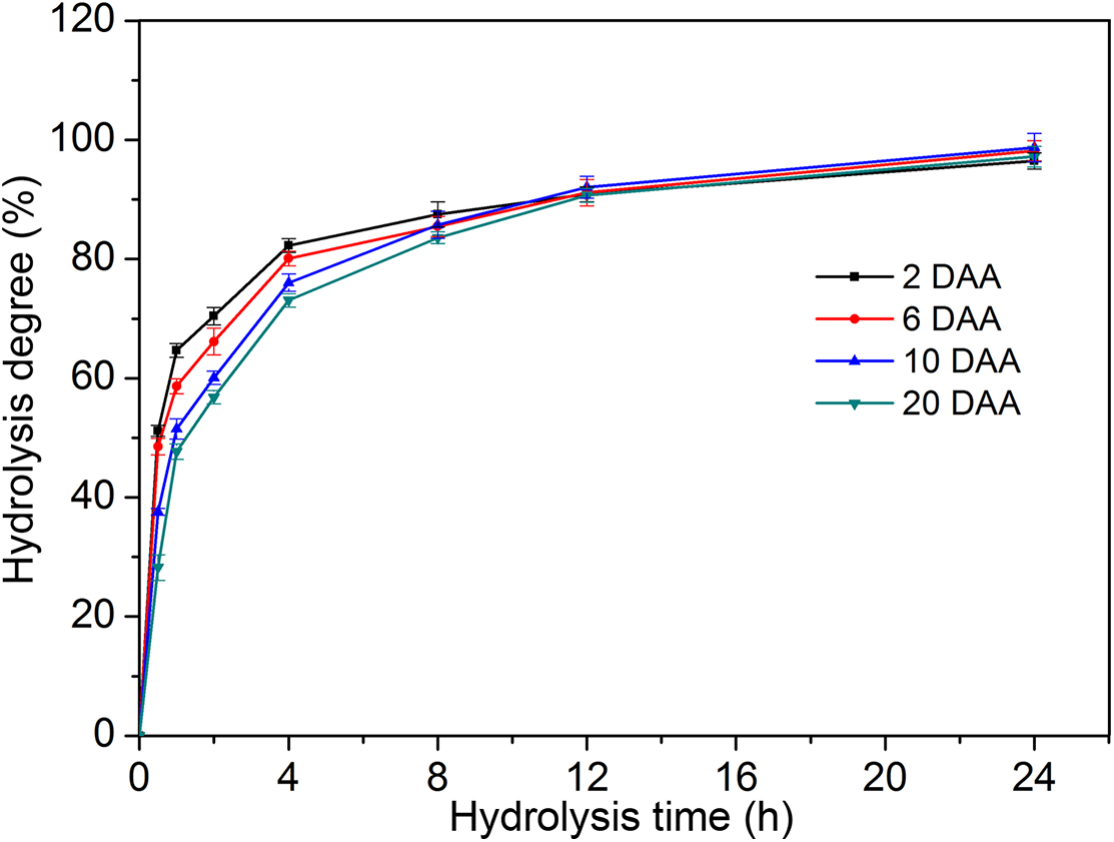
Degrees of enzymolysis of PSGs at 2, 6, 10, and 20 DAA.

Fig 7 shows the degree of enzymolysis of PSGs at 2, 6, 10, and 20 DAA. All PSGs exhibited rapid enzymolysis from 0 h to 4 h and slow enzymolysis from 4 h to 24 h. From 0 h to 4 h, the degree of enzymolysis of PSGs varied in the following order: 2 DAA>6 DAA>10 DAA>20 DAA (Fig 7). At 24 h, all of the PSGs presented high degrees of enzymolysis: 97% at 2 DAA, 98% at 6 DAA, 99% at 10 DAA, and 97% at 20 DAA.

### Expression analysis of *AGPase*, *GBSS II*, and *AMY* genes during wheat pericarp development

**Fig 8:**
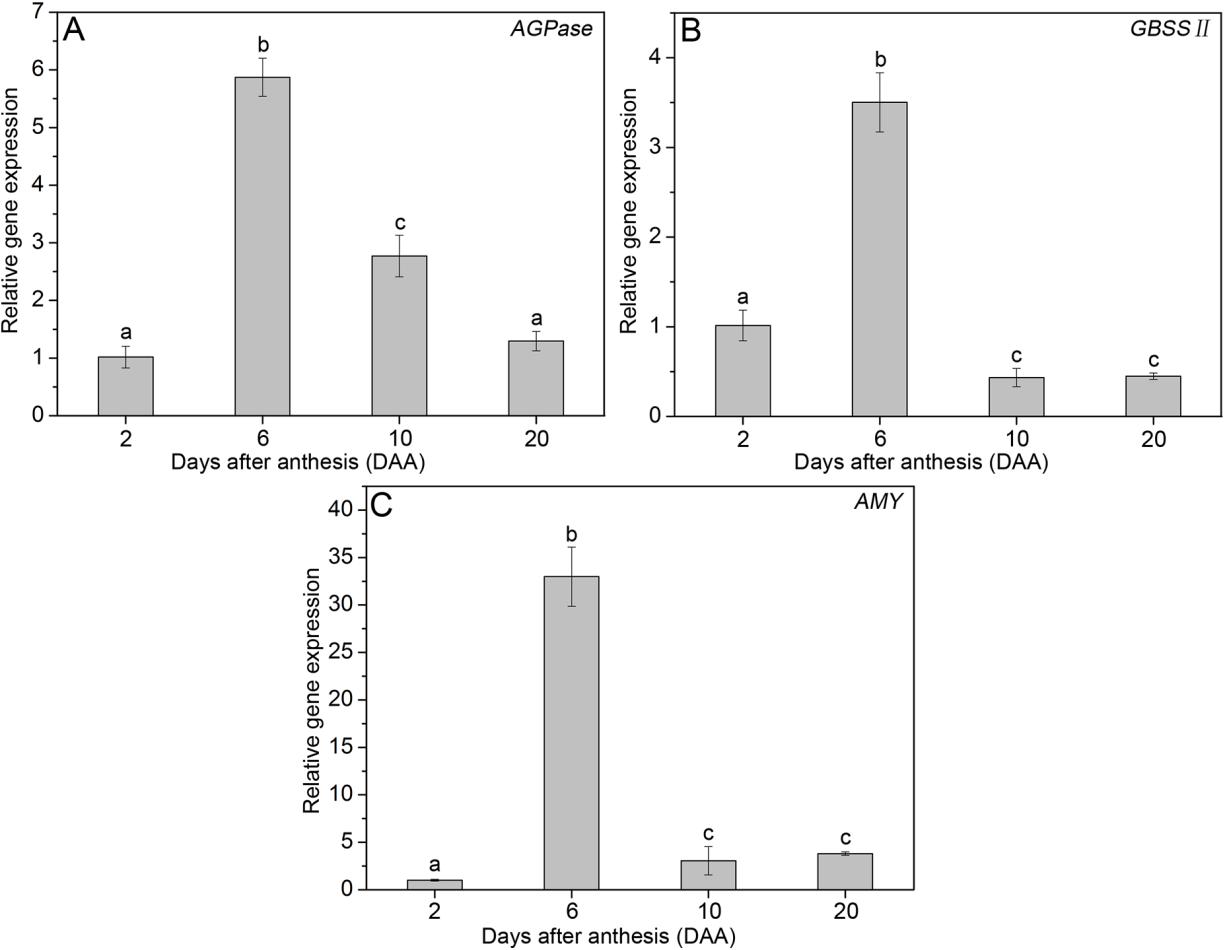
Relative gene expression levels of the genes *AGPase* (A), *GBSS II* (B) and *AMY* (C) in wheat pericarp at 2, 6, 10, and 20 DAA. Significant differences (P ≤ 0.05) between different DAAs are indicated by lowercase letters.

The relative expression levels of the genes *AGPase*, *GBSS II*, and *AMY* in wheat pericarp at different developmental stages (2, 6, 10, 20 DAA) were examined by qPCR, and patterns of relative gene expression are shown in Fig 8. For the *AGPase* gene, expression increased from 2 DAA to 6 DAA and gradually decreased thereafter (Fig 8A). For *GBSS II*, peak transcript level appeared at 6 DAA (Fig 8B). *AMY* showed very low relative expression at 2 DAA and maximum relative expression at 6 DAA. Relative expression from 10 DAA to 20 DAA was significantly lower than that at 6 DAA (Fig 8C).

## Discussion

### Accumulation and degradation mechanism of PSGs

PSGs accumulate in both amyloplasts and chloroplasts, which is in line with the findings reported by Yu et al. (2015) [18]. Similar results have also been reported in other cereal crop caryopses, such as barley [27], rice [16, 28] and sorghum [7]. The PSGs are also found in two main regions of wheat pericarp, namely, cross cells and MCVP. This may be associated with the biological characteristics of this species. As reported by Zheng and Wang (2015) [3], accumulate only in amyloplasts in this crop. The nutrient transfer tissues of wheat are much less advanced than those of maize [3, 29]. As such, the chlorophyll-containing layer in wheat caryopsis may produce photosynthetic products and provide extra nutrients for pericarp development at early developmental stages; during this time, nutrient transfer tissues are not well differentiated [30].

PSGs began to appear at 6 DAA and then gradually disappeared until 20 DAA. This phenomenon may be explained in two ways: (1) from 6 DAA to 20 DAA, endosperm cells undergo a period of material enrichment, including protein body and starch granule synthesis [31] (Fig 3). The number and volume of endosperm cells rapidly increase during this period, but the size of the caryopsis increases only slightly (Fig 3). Extensive endosperm cell expansion causes mesocarp cells to deform, crack, and degrade. (2) The *AMY* gene shows significantly higher relative expression at 6 DAA than at 2 DAA. Laethauwer et al. (2013) [32] reported that increased α-amylase activity was associated with increased *AMY* gene expression in winter types of triticale. Thus, the *AMY* gene product α-amylase likely catalyzes the hydrolysis of starch granules from 6 DAA to 20 DAA.

### Changes in total starch, soluble sugar and amylose contents

Dynamic changes in total starch and soluble sugar contents reveal that decreases in soluble sugar contents from 2 DAA to 6 DAA may support starch synthesis, which results in increases in total starch content. These results are consistent with the relative expression of the *AGPase* gene, which activates the first step of starch biosynthesis and shows increases from 2 DAA to 6 DAA and decreases from 6 DAA to 20 DAA. From 6 DAA to 10 DAA, soluble sugar contents increased rapidly and starch granule hydrolysis could be observed. From 10 DAA to 20 DAA, both total starch and soluble sugar contents decreased, which indicates that these carbohydrates are not only responsible for pericarp survival but also provide additional nutrients for endosperm growth. Similar ideas have been proposed previously by Yu et al. (2015) [18] and Zhou et al. (2009) [17].

GBSS is the only starch synthase that is found exclusively within starch granules and controls amylose synthesis [33]. GBSS II is an isoform of GBSS expressed in wheat pericarp [5]. The *GBSS II* gene showed higher relative expression from 2 DAA to 6 DAA than at later stages, indicating that the activity of amylose synthesis was greater at early stages than that at later stages. When starch was hydrolyzed by amylase, the amorphous region containing amylose was degraded first, followed by the crystalline region containing amylopectin molecules [34]. This may account for the lower amylose contents observed in subsequent days (10 and 20 DAA).

### Morphological and structural changes in PSGs

PSGs were found to have not only large granule sizes but also rough granule surfaces, which could be caused by α-amylase attack. When hydrolyzed by α-amylase, the degree of enzymolysis of PSGs followed the order: 2 DAA>6 DAA>10 DAA>20 DAA. These results could be due to the ratio of surface area to volume of granules, since previous studies demonstrated that starch granules with smaller sizes show higher degrees of enzymolysis [35]. In this study, PSGs were almost completely hydrolyzed by α-amylase, with degrees of enzymolysis ranging from 96% to 99% at 24 h. When hydrolyzed by α-amylase using the same method adopted in the present study, degrees of enzymolysis of about 82% and 7% were obtained at 72 h for normal rice starch and potato starch, respectively [36]. Incomplete starch degradation may be attributed to the resistant-starch content of the sample under study [37]. As temporary energy storage materials, the high degree of enzymolysis of PSGs may provide more nutrients for pericarp metabolism.

XRD and ATR-FTIR are advanced technologies that have been successfully used to study long-range ordered structures of whole granules and short-range ordered structures of external regions of starch granules, respectively [22, 38]. The relative degrees of crystallinity (long-range ordered structure) of PSGs were significantly higher at 10 and 20 DAA than at 2 and 6 DAA. The ATR-FTIR absorption bands at 1045 and 1022 cm^−1^ are associated with crystalline lamellar and amorphous structures, respectively, while the band at 995 cm^−1^ is attributed to the bending vibrations of C–OH bonds [39]. The IR ratios of 1045 cm^−1^/1022 cm^−1^ and 1022 cm^−1^/995 cm^−1^ are considered quantitative indices for evaluating the short-range ordered structures of starch granules [23]. In this study, ATR-FTIR revealed a lack of significant differences in short-range ordered structures in the external regions of these granules. These results demonstrate that structural changes in PSGs take place only in the internal regions of granules rather than external regions.

## Supporting Information

S1 Table The list of detected sequences of *ADP-glucose pyrophosphorylase*, *granule-bound starch synthase II*, *α-amylase* and *ADP-ribosylation factor* gene. Additional supporting information may be found in the online version of this article at the publisher’s web-site.
(DOCX)
