# Supplementary material for: Systematic Analysis of Pericarp Starch Accumulation and Degradation during Wheat Caryopsis Development
Source: PLoS One. 2015 Sep 22;10(9):e0138228. doi: 10.1371/journal.pone.0138228 (PMC4578966; doi:10.1371/journal.pone.0138228)
Supplement: S1 Table — Additional supporting information may be found in the online version of this article at the publisher’s web-site. (DOCX) [file pone.0138228.s001.docx]

**S1 Table. The list of detected sequences of *ADP-glucose pyrophosphorylase*, *granule-bound starch synthase II*, *α-amylase* and *ADP-ribosylation factor* gene**

| Gene | Sequence |
| --- | --- |
| *ADP-glucose pyrophosphorylase* | CGGCAATGGATGTGCCTTTGGCATCTAAAACATTCCCCTCGCCCTCACCTTCCAAGCGTGAACAATGCAACGTTGATGGCCATAAGAGCTCATCGAAGCATGCAGATCTCAATCCACATGCTAATGATAGTGTTCTCGGAATTATTCTTGGAGGTGGTGCAGGGACTAGATTGTATCCCCTGACGAAGAAGCGTGCGAAGCCTGCAGTGCCACTGGGTGCCAACTACAGGCTTATTGATATTCCTGTCAGTAATTGTCTGAACAGCAACATATCAAAGATCTATGTGCTCACGCAGTTCAACTCAGCTTCTCTTAATCGTCATCTCTCACGAGCCTATGGGAGCAACATTGGAGGTTACAAGAATGAAGGATTTGTTGAAGTCCTTGCTGCACAGCAGAGCCCAGATAACCCTGACTGGTTTCAGGGTACTGCAGATGCTGTAAGGCAGTACTTGTGGCTATTCGAGGAGCATAATGTTATGGAGTATCTAATTCTTGCCGGAGATCACCTGTACCGAATGGACTATGAAAAGTTTATTCAGGCACACAGAGAAACAGATGCTGATATTACTGTTGCTGCCTTGCCCATGGATGAGGAACGTGCAACTGCATTTGGCCTTATGAAAATCGATGAAGAAGGGAGGATAATTGAATTCGCAGAGAAACCAAAAGGAGAACAGTTGAAAGCTATGATGGTTGATACGACCATACTTGGCCTTGACGATGCGAGGGCAAAGGAAATGCCTTATATTGCTAGCATGGGTATCTATGTTATTAGCAAACATGTGATGCTTCAGCTTCTCCGCGAGCAATTTCCTGGAGCTAATGACTTTGGAAGTGAGGTTATTCCTGGTGCAACTAGCACTGGCATGAGGGTACAAGCATACCTATACGATGGTTACTGGGAAGATATTGGTACAATTGAGGCATTCTATAATGCAAATTTGGGAATTACCAAAAAGCCAATACCCGATTTCAGTTTCTATGACCGTTCTGCTCCCATTTACACACAACCTCGACACTTGCCTCCTTCAAAGGTTCTTGATGCTGATGTGACAGACAGTGTTATTGGTGAAGGATGTGTTATTAAAAACTGCAAGATACACCATTCAGTAGTTGGACTCCGGTCCTGCATATCTGAAGGGGCAATAATAGAGGACACATTGCTAATGGGTGCGGACTACTATGAGACTGAAGCTGATAAGAAACTCCTTGCTGAAAAAGGTGGCATTCCCATTGGTATTGGAAAGAATTCACACATCAAAAGAGCAATAATTGACAAGAATGCTCGTATTGGAGATAACGTGATGATAATCAATGTTGACAATGTTCAAGAAGCGGCAAGGGAGACAGATGGATATTTCATCAAAAGTGGCATTGTAACTGTGATCAAGGATGCTTTACTCCCTAGTGGAACAGTCATATGAAGC |
| *granule-bound starch synthase II* | ATGGGTTCCATTCCTAATTATTGTTCTTATCAAACAAACAGTGTTGGTTCACTGAAACTGTCACCTCACATCCAATTCCAGCAATCTTGTAACAATGGAGTTATGTTTGTATCCATGCGGAATAAGACACAGCTGGCCAAAAGAAGAGCCACAAATTATGGAACTCATCCTAATTCTAGTAGGACTCCTGCACCTATCGTATGTTCAACTGGAATGCCCATAATTTTCGTTGCCACTGAAGTGCACCCATGGTGCAAAACTGGAGGCCTTGGTGATGTCGTAGGAGGATTGCCCCCAGCTCTGGCTGCAATGGGGCACCGGGTTATGACGATAGCTCCTCGCTATGATCAGTACAAGGATACATGGGATACAAATGTTCTTGTTGAGGTAATTGTTGGTGACAGAACAGAAACAGTGCGCTTTTTTCACTGCTACAAAAGGGGAGTTGATCGTGTTTTTGTTGATCATCCTATGTTTCTTGAGAAGGTATGGGGCAAAACTGGATCAAAATTGTACGGGCCTACCACTGGAACAGACTTCCGAGATAACCAGTTACGGTTCTGCCTTTTGTGCCTTGCTGCATTGGAGGCTCCGAGGGTTCTTAATCTCAATAATTCTGAATACTTCTCTGGACCATATGGAGAAAATGTTGTCTTCGTTGCAAATGACTGGCACACTGCAGTTTTGCCATGCTATTTGAAGAGCATGTATAAGCAAAATGGAATTTATGAGAATGCCAAGGTTGCTTTCTGCATTCACAATATCGCCTATCAGGGCAGATTTCCCAGAGCGGACTTCGAACTTCTTAATTTACCTGAAAGTTTCATGCCGTCATTTGATTTTGTTGATGGGCATGTTAAGCCAGTAGTAGGGAGAAAGATTAACTGGATGAAGGCAGGGATCACTGAGTGTGACGTGGTCCTTACAGTTAGTCCACATTATGTCAAAGAACTCACTTCTGGCCCAGAGAAAGGTGTTGAGTTGGATGGCGTCCTTCGTGCAAAGCCTCTTGAAACTGGAATTGTAAATGGAATGGATGTTGTTGATTGGAATCCAGCAACAGATAAGTACATCAGTGTCAAATACAATGCAACAACGGTGGCAGAAGCAAGAGCTCTCAATAAAGAAATACTGCAAGCTGAAGTTGGATTGCCAGTGGACTCTAGCATACCTGTTATAGTTTTCATTGGACGTCTTGAAGAACAGAAAGGGTCAGACATACTAATTGCAGCCATACCGGAGTTTCTCGAGGAGAATGTTCAGATAATTGTTCTCGGCACAGGGAAGAAGAAAATGGAGGAGGAACTGATGCTGCTAGAAGCGAAGTACCCACAGAATGCCAGAGGCATAGCAAAATTCAATGTCCCATTGGCGCACATGATGTTCGCCGGGGCTGATTTCATCATTGTTCCAAGTAGGTTTGAGCCATGTGGCCTCATCCAATTGCAAGGGATGAGATATGGAGTGATTCCCATCTGTTCATCCACCGGAGGACTTGTTGACACGGTGAGGGAGGGTGTCACCGGATTCCACATGGGTTCGTTCAATGTCGAGTTTGAAACCGTCGATCCAGCAGATGTCGCGGCAGTCGCTTCGAATGTCACACGAGCTCTGAAACAGTACAAAACACCGTCGTTCCACGCAATGGTTCGGAATTGCATGGCGCAGGACCTATCTTGGAAGGGACCGGCAAAGAAGTGGGAGGAGGCACTTCTTGGCCTAGGAGTCGAGGGAAGTCAGCTGGGCATCGAGGGCGAGGAGATCGCTCCACTTGCGAAACAGAATGTGGCCACTCCCTGA |
| *α-amylase* | GGCAAGCACTCTGCTACTCTCTGTGGCCTTCTTGTTGTTGTGCTTTGTCTCGCCTCCAGCTTAGCACAGGCTCAAATTCTTTTCCAGGGGTTTAACTGGGAATCATGGAAGACACAAGGCGGCTGGTACAAGTTCATGCAAGGGAAGGTGGACGACATCGCCAGCACCGGCGCCACCCACGTCTGGCTCCCCCCGCCGTCGCAGTCCGTGTCGCCGGAGGGCTATCTGCCGGGGCAGCTCTACAATCTTAACTCCAAGTACGGCAGCGGGGCTGACCTCAAGTCACTGATCAAGGCGTTCCGCAGCAAGAACATCTCATGCGTCGCCGACATCGTCATCAACCACCGCTGCGCCGACAAGAAGGACGGTCGCGGTGTCTACTGCATCTTCGAGGGCGGGACGTCCGACAACCGCCTCGACTGGGGCCCCGACGAGATCTGCAGCGACGACACCAAGTACTCCAACGGCCGCGGCCACAGAGACACCGGCGGTGGCTTCGACGCCGCGCCCGACATCGACCACCTCAACCCGCGGGTGCAGAGGGAGCTCTCCGCCTGGCTCAACTGGCTCAAAACCGACCTCGGCTTCGTTGGATGGCGCCTCGACTTTGCCAAGGGATACTCCGCGGCCATGGCCAAGATCTACGTTGACAACAGCAAGCCGTCGTTCGTGGTTGGTGAGCTCTACGACAGGGACCGACAGCTGCTCGCGAACTGGGTGCAGGGCGTCGGCGGGCCGGCCACGGCGTTTGACTTCCCCACCAAGGGTGTGCTCCAGGAGGCCGTGCAGGGCGATCTTGGGAGGATGCGTGGCAACGGCGGCAAGGCACCCGGTCTGATCGGGTGGATGCCCGAGAAGACCGTCACGTTCATCGACAACCACGACACCGGGTCGACCCAGAGGCTCTGGCCGTTCCCCTCAGACAAGGTCATGCAGGGCTACGCCTACATCCTCACACACCCGGGCATACCATGCCTCTTCTACGACCACGTGTTCGACTGGAAGCTGAAGCAGGAGATCACCGCACTGGCTAGGGTCAGGTCAAGGAACGGGATCCATCCGGGTAGCACACTGGATATCCTCAAGGCCGAGGGCGATCTCTATGTTGCCAAGATTGGGGGCAAGGTTATAACCAAGATCGGGTCAAGATACAATATTGGCAACAACGTGATCCCCTCGGGTTTCAAGATTGCGGCTAAAGGCAACAACTACTGTGTCTGGGAGAAGAGCGGCCTCTGA |
| *ADP-ribosylation factor* | GCTCACGTTCACCAAGCTGTTCAGCCGCCTGTTCGCCAAGAAGGAGATGCGGATCCTGATGGTGGGTCTCGACGCCGCCGGAAAGACCACCATCCTCTACAAGCTCAAGCTCGGCGAGATCGTCACCACCATCCCCACCATCGGGTTCAATGTTGAAACTGTGGAGTACAAGAACATCAGCTTCACTGTCTGGGATGTCGGGGGTCAGGACAAGATCAGGCCACTGTGGAGGCATTACTTCCAGAACACACGGGGTCTCATCTTTGTTGTGGACAGCAACGATAGGGACCGTGTTGTTGAGGCAAGGGATGAGCTCCACAGGATGCTGAATGAGGATGAGTTACGTGATGCTGTGCTGCTTGTGTTTGCTAACAAGCAAGATCTTCCAAATGCCATGAATGCTGCTGAGATCACTGATAAGCTTGGCCTGCACTCCCTTCGCCAGCGACACTGGTACATCCAGAGCACTTG |
